# “XXIst century odyssey of Medicine” stem cells and their future

**DOI:** 10.3389/fphys.2013.00250

**Published:** 2013-09-10

**Authors:** J. Bouquet de la Jolinière, A. Feki

**Affiliations:** Department of Reproductive Biology and Obstetrics and Gynecology, Fribourg HospitalFribourg, Switzerland

**Keywords:** pluripotent stem cells, iPS cells, IVF stem cells, embryonic stem cells

The last century is undoubtedly the one of discoveries which have changed the destiny of humanity in a very short time. Quantum physics have opened the door of nanotechnology, information technologies, and their multiple applications in medicine. The discovery of penicillin and antibiotics has helped with the development of a safe and aseptic surgery. Moreover, microbiology, molecular biology, genetics, and the human genome sequencing have given us knowledge and wonderful tools to help to understand the mechanisms that rule life-sciences. They have also given us the technology to study gene transmission and their expression. Thus, it is nowadays possible to treat many diseases, for example in the field of oncology. Last, organ transplantation is part of these medical innovations.

During these discoveries, especially those related to IVF, ethical problems have arisen during the cloning of embryos, attempt of embryos manipulation, and embryonic stem cells derivation for therapeutic use. Pluripotent stem cells and deriving induced pluripotent stem cells represent the greatest medical advance of this century and were awarded the Nobel Prize for Medicine in 2012 (Yamanaka, 2012). Therefore, hope has arisen in several areas like neurology, cardiology, tooth regeneration, and reproductive medicine.

If it is now possible to consider repairing organs by autologous grafting like after myocardial infarct, or spinal cord injury (Hibaoui and Feki, 2012), the approach using pluripotent stem cells in reproductive medicine raised ethical and scientific questions. Obtaining functional sperm or oocytes from skin derived-iPS cells may lead to the disappearance of the term of infertility, and IVF programs would benefit from this development. Stem cell field also offers other possibilities like for pharmacology of drug testing, disease modeling, and the discovery of new gene functionalities.

The reviews of this special issue treated several of the aforementioned fields. This century will be the one of stem cell biology and genetics: the humankind will be attracted by findings of this science. It will not create but will try to repair, to live longer, and to rejuvenate. If Einstein believed that genius is intuition, we have enough models at our disposal to take over the following meaningful sentence of Master Eckhard who said: “it is not our actions that sanctify us, but, we who sanctify our actions.”
